# Martini Mapper: An Automated Fragment-Based Mapping Algorithm for Developing Coarse-Grained Models within the Martini 3 Framework

**DOI:** 10.1021/acs.jcim.5c02903

**Published:** 2026-04-28

**Authors:** Kevin V. Bigting, Shubhadeep Nag, Yaxin An

**Affiliations:** † Division of Computer Science and Engineering, 5779 Louisiana State University, Baton Rouge, Louisiana 70803, United States; ‡ Department of Chemical Engineering, 5779 Louisiana State University, Baton Rouge, Louisiana 70803, United States

## Abstract

Coarse-graining (CG) reduces molecular details to extend the time and length scales of molecular dynamics simulations to microseconds and micrometers. However, the CG approaches have long been limited by the difficulty of constructing both accurate and transferable models efficiently, considering the large diversity of chemical structures of materials. Among CG force fields, Martini is the most widely used, as it retains essential chemical features while offering substantial computational efficiency. Its most recent version, Martini 3, expands chemical resolution through a much broader bead set, particularly for small molecules. However, this flexibility also complicates the mapping of organic molecules because of context-dependent rules and the lack of standardized procedures. To address this issue, we present an automated framework that builds Martini 3 models directly from SMILES (Simplified Molecular Input Line Entry System) strings by combining a curated bead dictionary with a hierarchical, rule-based algorithm and molecule-specific bonded parameters. Our framework, Martini Mapper https://github.com/eliobaby/Martini_mapper, generated Martini 3 models for 6280 molecules across six chemically diverse data sets, including 1689 systems with bond/angle parameters and additional large systems mapped at the topological level. A curated subset of 1075 mapped structures was benchmarked using transfer free energies in hydrated octanol, hexadecane, and chloroform from water against reference data wherever available. We further examined the benchmark with structural validation via SASA, yielding good agreement with experimental and atomistic reference data. The workflow can also map large molecules containing up to 172 heavy atoms, exceeding the capabilities of existing automated approaches. Our framework, therefore, enables systematic and scalable Martini 3 structures for high-throughput simulations relevant to drug discovery and materials design.

## Graphical abstract

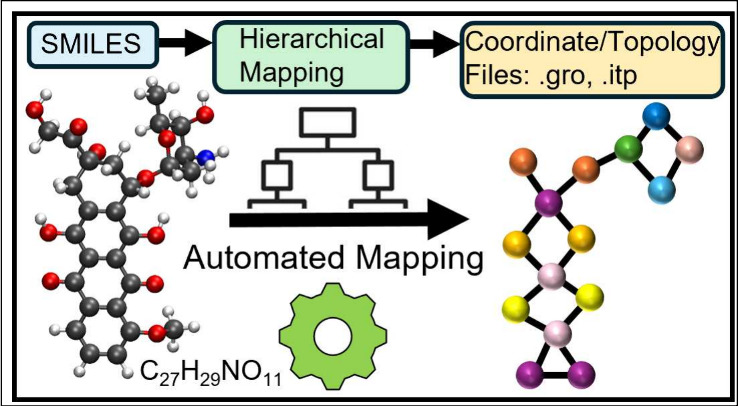

## 1 Introduction

Molecular dynamics (MD) simulations were introduced in the early 1960s^1^ to translate classical mechanics into predictive models of atomic motion, initially targeting simple liquids and crystalline solids.^2^ Over the past five decades, MD has evolved into a central technique in chemical and biological physics,^3,4^ enabling the resolution of molecular processes inaccessible to analytical theory or direct observation. By integrating empirical or quantum-derived force fields with Newtonian dynamics,^5^ MD provides access to atomically detailed trajectories over nanosecond to microsecond time scales.^6,7^ This capability makes MD indispensable for understanding structure–function relationships in biomolecules, molecular recognition, and soft matter behavior.^8,9^ Its predictive relevance has steadily increased, particularly in contexts where experimental resolution is limited or transient intermediates dominate, such as protein conformational switching, allosteric modulation, or lipid membrane remodeling.^10,11^ In recent applications, MD has been leveraged to elucidate drug-binding kinetics,^12^ refine cryo-EM structural models,^13^ and identify cryptic pockets in viral proteins,^14^ including the SARS-CoV-2 spike.^15,16^ Critically, with the increase in computational power and advancing integration techniques, MD is no longer merely descriptive; it increasingly guides experimental design, suggesting hypotheses, validating interpretations, and accelerating discovery across molecular sciences.^17–19^

The focus of molecular simulations has always been operating across a hierarchy of spatial and temporal resolutions, with each level balancing computational cost and chemical detail. At the all-atom (AA) level, each atom, including hydrogens, is treated as an explicit interaction site, enabling rigorous representation of directional interactions and conformational energetics. These force fields are parametrized via hybrid protocols incorporating quantum mechanical data and thermophysical observables, and are routinely benchmarked against experimental structures and time-correlation functions.^20–22^ United-atom (UA) models introduce a systematic reduction by integrating nonpolar hydrogens into their parent heavy atoms, reducing the number of degrees of freedom while retaining molecular topology and core thermodynamic adherence.^23,24^

While UA models reduce computational cost modestly, many biologically or technologically relevant processes span length- and time-scales that remain inaccessible even at this intermediate resolution. To overcome these barriers, coarse-grained (CG) representations extend atomistic resolution reduction by mapping groups of atoms into single interaction sites or beads, thereby lowering the number of degrees of freedom and smoothing the underlying energy landscape.^25–27^ This abstraction enables simulations of large molecular assemblies and slow collective processes, granting access to mesoscopic length and time scales beyond the reach of all-atom approaches.^28–32^ Although the reduction in chemical specificity is an inherent trade-off, modern CG models, particularly those developed within the Martini framework have proven capable of capturing mesoscale organization, emergent dynamics, and key thermodynamic observables with remarkable fidelity. Recent advances demonstrate predictive accuracy in contexts ranging from protein–ligand binding and lipid self-assembly to biomolecular condensates.^33,34^ When rigorously parametrized and validated against atomistic simulations or experimental benchmarks, CG models thus function not merely as simplified surrogates but as powerful, resolution-adaptive tools within multiscale simulation workflows that bridge molecular detail with emergent material and biological phenomena.

Among CG force fields, Martini is the most widely adopted due to its balance between transferability, accuracy, and computational efficiency. The original Martini 2 force field became a standard for simulating lipids, proteins, and small molecules, offering a chemically intuitive four-to-one mapping scheme.^35–37^ However, its limited bead vocabulary often led to oversimplifications, particularly for polar and aromatic systems.^38^ Martini 3 addresses these issues by introducing a refined and expanded bead set, enhanced size resolution (tiny, small, regular), and improved mapping rules that capture chemical diversity with greater fidelity.^39,40^ At the same time, Martini 3 exhibits increased sensitivity to bonded interaction parametrization and molecular volume consistency, making accurate bond, angle, and structural definitions critical for stable and transferable models. This increased resolution improves accuracy but also introduces complexity in mapping, especially for small molecules with varied functional groups.^41^ As a result, manual mapping becomes a bottleneck, motivating the development of automated, rule-based approaches to fully leverage Martini 3’s capabilities at scale.^42–45^

A range of methodologies has been developed to automate or optimize atomistic-to-coarse-grained mapping, using rule-based, graph-theoretic, and machine learning (ML) approaches. For example, the Deep Supervised Graph Partitioning Model (DSGPM)^46^ and MolCluster^47^ use graph neural networks in supervised and unsupervised settings, respectively, to automate coarse-grained mapping as data-driven alternatives to manual approaches. Webb et al.^48^ proposed the Graph-Based Coarse-Graining (GBCG) method, systematically generating mappings via edge contractions on molecular graphs. Zhong et al.^47^ extended this by integrating a neural architecture with optimizer flexibility in AMOFMS, which enables both bottom-up and top-down parametrization. Potter et al.^43^ developed an automated mapping algorithm specific to the Martini force field, combining graph analysis with heuristics for ring handling and membrane partitioning benchmarks. Bereau and Kremer^49^ proposed a protocol for automatic Martini parametrization, validated on hydration and partition free energies. Recent advances in bonded parameter automation include PyCGTOOL,^50^ which derives equilibrium bond and angle values directly from atomistic trajectories, and Bartender,^51^ which employs quantum mechanics-based molecular dynamics to extract Martini 3 bonded terms with improved numerical stability. These developments build upon advances in extended tight-binding (xTB) quantum chemistry methods,^52^ which enable fast and broadly applicable semiempirical atomistic simulations across diverse chemical systems. In parallel, force-field level extensions such as GFN-FF further demonstrate the feasibility of fully automated construction of molecular force-field terms across large chemical spaces.^53^ Together, these approaches underscore the importance of systematic, molecule-specific bonded parametrization strategies in multiscale modeling workflows. Wang and G’omez-Bombarelli^54^ employed variational autoencoders to simultaneously learn CG variables and their backmapping, while Zhang et al.^55^ introduced DeePCG, a deep learning model preserving many-body correlations. Rudzinski and Noid^56^ provided a theoretical basis for evaluating CG mappings using iterative g-YBG theory, and Mahajan and Tang^57^ presented an automated framework for polyethylenimine mapping under Martini with validation against experimental observables.

Other efforts contribute tools for CG system construction and visualization,^58^ highlight statistical inconsistencies in Martini models,^59^ or discuss broader applications in macromolecular modeling.^60^ Although these methods advance the CG mapping landscape, key challenges persist. Most models focus on either fixed-resolution mapping or specific chemical classes, and few generalize across chemically diverse small molecules. Crucially, the expanded chemical vocabulary in Martini 3 amplifies mapping ambiguity, especially in aromatic, branched, or heteroatom-rich systems. Existing ML frameworks often depend on curated training data or fail to yield directly simulation-ready topologies.^61^ Moreover, while some approaches address mapping prediction, they do not integrate rule-based validation or ensure reproducibility across runs.^62,63^ These limitations collectively motivate us to build a unified, rule-driven framework capable of mapping arbitrary molecules into Martini 3 representations with full automation, extensibility, and physical consistency. Recent progress using the Martini 3 force field for small molecules includes Auto-MartiniM3,^45^ which demonstrates strong scalability and performance on small chemical systems. However, systematic integration of molecule-specific bonded parameter extraction and validation across diverse chemical spaces remains an open challenge. Furthermore, it is to note that although the Martini 3 model construction is supported by comprehensive guidelines and well-documented best practices,^64,65^ applying these conventions systematically across large and chemically diverse data sets remains nontrivial in high-throughput settings.

In this work, we present an automated framework that generates Martini 3 coarse-grained models of small molecules with a range of heavy atoms from 2 to 172, directly from canonical Simplified Molecular Input Line Entry System (SMILES) strings. The motivation of Martini_Mapper is to formalize and automate the application of these existing rules into a reproducible and extensible workflow suitable for large-scale molecular screening. The framework combines a curated bead-mapping dictionary with a hierarchical, rule-based mapping algorithm and integrates molecule-specific bonded parameters derived from xTB-based ensemble sampling. The algorithm automatically identifies rings, side chains, and functional groups through structural analysis, followed by prioritized hierarchical bead assignment that enforces molecular symmetry. Across six chemically diverse data sets, Martini_Mapper generates 6280 mapped topologies, including 1689 systems for which bonded parameters were derived from xTB-based molecular dynamics sampling. To evaluate predictive fidelity, we benchmark the generated models against experimentally measured transfer free energies in multiple solvent systems (hydrated octanol, hexadecane, and chloroform), together with structural validation via solvent-accessible surface area (SASA) comparisons against atomistic reference structures. These validations demonstrate reasonable agreement across chemically diverse systems. Our framework produces GROMACS-compatible coordinate and topology files in a fully reproducible and simulation-ready form, thereby enabling scalable high-throughput model construction. Finally, we report current limitations and outline future directions of Martini_Mapper. In essence, our model establishes a reproducible foundation for data-driven coarse-grained modeling, facilitating applications in drug discovery, polymer–drug assembly, and biomolecular condensates.

## 2 Algorithmic Framework for Automated Coarse-Grained Mapping

The automated algorithmic framework is a fragment-based method developed for mapping atomic fragments to coarse-grained beads. The first step in this framework is constructing a bead dictionary, which serves as a reference that links specific molecular fragments to predefined coarse-grained bead types. Each bead entry corresponds to a chemically meaningful unit, such as an alkyl chain, an aromatic ring fragment, or a functional group. The mapping process consists of three key steps: input processing, mapping, and output generation, which is shown in the flowchart in Figure 1. The input processing involves preprocessing the SMILES string of a molecule.^66^ This string encodes the full atom-level structure of the molecule, including its connectivity, ring closures, and branching patterns. From this representation, the framework extracts the topological information necessary to identify chemically distinct fragments and prepare required matrices (e.g., adjacency/property matrices) for mapping. Once the input is processed, the framework applies a hierarchical rule-based mapping algorithm to assign beads to different parts of the molecule. The mapping rules are organized so that the mapping starts with the most structurally constrained regions, which in our framework typically ring systems, and then proceeds to nonring fragments, including chains and side groups. This layered approach allows the framework to ultimately generate Martini 3-compatible coarse-grained models (including the summary, coordinates, and topology) in a fully deterministic and reproducible manner. We will explain each of these steps with specific examples in detail below.

**Figure 1:**
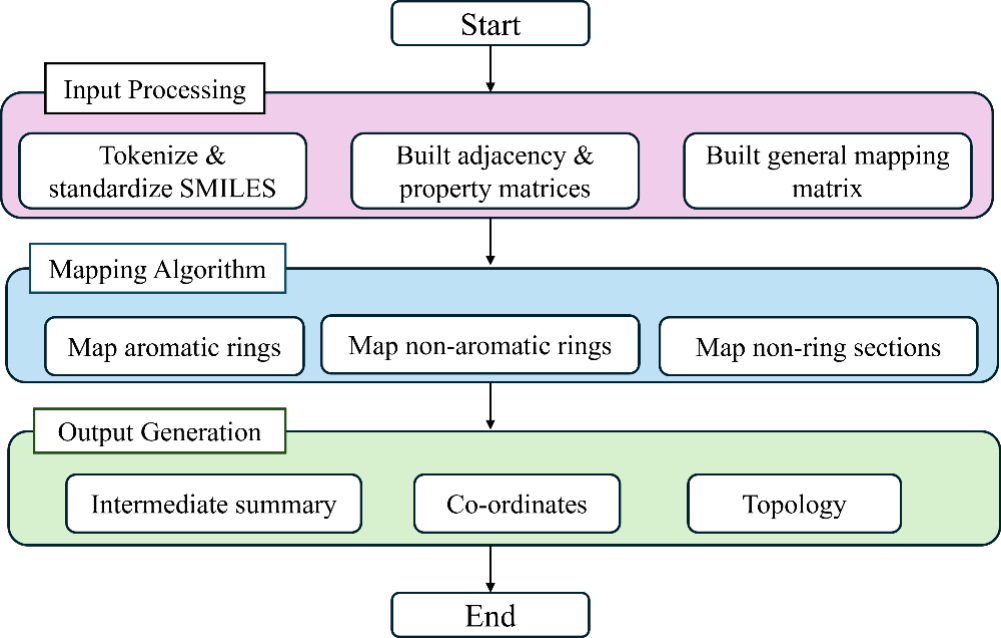
Flowchart of our automated coarse-grained mapping pipeline. The process begins with SMILES input, proceeds through preprocessing (tokenization, graph construction, and mapping array generation), applies the hierarchical bead assignment algorithm, and outputs simulation-ready coordinate (.gro) and topology (.itp) files.

### 2.1 Building Literature-Based Building Block Table (LBBT)

To construct the initial bead dictionary, we integrated two complementary sources from the Martini 3 framework and compiled them into a literature-based building block table (LBBT). The first source is the list of all the 90 small molecules reported in the Martini small molecule data set,^41^ where each entry corresponds to a chemically defined substructure derived from validated coarse-grained models. These fragment-level entries are chemically precise with respect to the influence of neighboring atoms and topological features. The second source, that we use is the supplementary Table 24 from the Martini 3 force field,^39^ which lists default bead assignments for generic chemical groups without contextual detail. Here, the examples include: (a) alcohol mapped to P1, (b) carboxylic acid to P2, (c) phenol to N6, (d) linear alkane to C1, etc. While this table lacks fragment specificity, it covers the most common functional groups.

As a third source, we incorporated the benchmark data set reported in the Grünewald study,^67^ which introduces a line-notation designed to represent coarse-grained models across resolutions. This data set is chemically significant because it systematically aggregates many validated Martini mappings in machine-readable form, spanning diverse functional groups and bead types, thereby broadening coverage beyond fragment lists and generic group defaults. From this data set, we added fragment-to-bead correspondences that recur across mapped molecules, including: (a) sulfonamide-like fragments mapped to P4, which we represent with multiple chemically distinct sulfonamide fragments, (b) cyanamide mapped to P 4d, (c) carbon disulfide mapped to C6, and (d) carbon dioxide mapped to SC6. These three sources were then merged to create a unified dictionary, LBBT, containing a total of 254 fragments.

### 2.2 Preprocessing of Molecular Structures from SMILES

As described before, our automated CG mapping framework begins with a SMILES representation of the target molecule. This preprocessing stage is performed in the following steps that transform a one-dimensional text-based input into a structured data model suitable for rule-based bead assignment within the Martini 3 force field beads.

(i) **SMILES tokenization and standardization:** The canonical SMILES string is parsed into a discrete sequence of tokens, where each token represents either an atom or a structural modifier such as a ring closure index. Tokenization ensures that equivalent structures yield identical token sequences, while standardization removes stereochemical and isotopic annotations not required at CG resolution. This reduction simplifies the mapping space, allowing consistent pattern recognition and minimizing ambiguity during rule matching. For illustration, the methyl 3-furancarboxylate molecule (o1ccc­(C­(=O)­OC)­c1) is tokenized into an ordered sequence corresponding to a five-membered aromatic ring containing one oxygen atom and a methoxycarbonyl (linear) side group (Figure 2).
(ii) **Construction of adjacency and property matrices:** From the tokenized representation, two primary data structures are built. The *property matrix* encodes atom-level attributes such as element type, aromaticity, ring membership, whether the atom lies at a fragment boundary (edge status), and hydrogen counts, which is used to distinguish fragments that share the same SMILES, e.g., separating primary, secondary, and tertiary amide/amine environments, local bonding environment such as whether an oxygen is in an ether (no hydrogen attached) or a hydroxyl (OH). The *connectivity matrix* encodes bond topology, with single bonds represented as 1.0, bonds in aromatic rings as 1.5, and double bonds as 2.0. For methyl 3-furancarboxylate, the four carbons and one oxygen are flagged as aromatic in a pentane ring, while the nonring linear chain is identified as a terminal substituent (see Table 1).
(iii) **General mapping array generation:** The adjacency and property information are combined into a general mapping array, a hierarchical grouping of atoms into structural sections such as aromatic rings, nonaromatic rings, and nonring fragments, encoded as “2”, “1”, and “0” in “Ring Status”, accordingly. Each entry includes atom indices, element types, connectivity context, and whether the atom is at the edge of a fragment. For methyl 3-furancarboxylate, the four carbons and one oxygen are flagged as aromatic in a five-membered ring, while the nonring linear chain is identified as a terminal substituent (see Table 1). The resulting mapping array partitions the molecule into two chemically distinct fragments: the five-membered aromatic ring (atoms 0–3, 8) and the nonring substituent (atoms 4–7). After defining these fragment boundaries, the framework specifies how atoms across fragments are connected, such as the single bond between atom 3 of the ring and atom 4 of the substituent, and atoms within a section are connected using inner connections, such as the connections between the atoms inside the ring, preserving complete molecular connectivity. For outer connections (connecting to other groups/sections), the bond entry is broken down into three parts: the index of the group in which the foreign atom resides (Outer Group), the index of the atom within that section (Outer Atom), and the bond order between the atoms (Outer Bond). For inner connections, only the bonded atom’s index within the section (Inner Atom) and the bond order (Inner Bond) are needed. This structure cleanly encodes both local and cross-section connections, as shown in Table 2.

**Figure 2:**
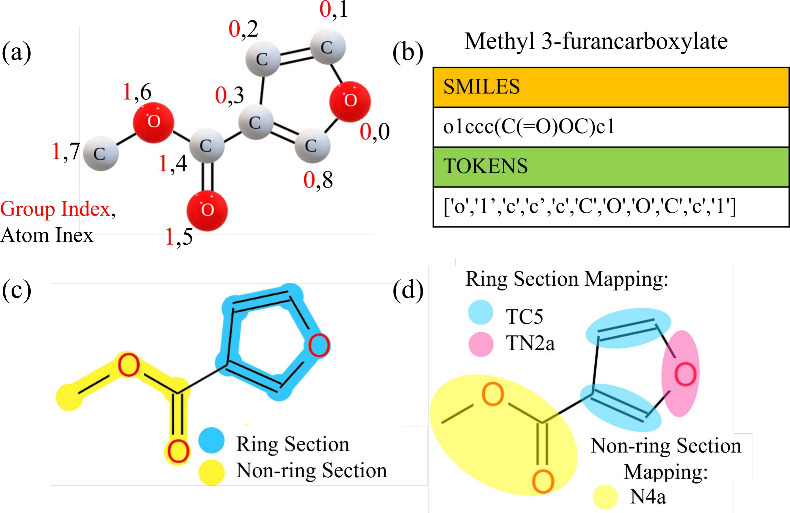
(a) Chemical structure of methyl 3-furancarboxylate. Note hydrogen atoms are not shown for clarity, but the number of hydrogen atoms attached to heavy atoms are counted (see Table 1). (b) Tokenization of the canonical SMILES string (o1ccc­(C­(=O)­OC)­c1) into atomic and structural symbols. Mapping Scheme: (c) Partitioning of the molecule into ring (blue) and nonring (yellow) sections according to the algorithm. (d) Final bead assignments: the aromatic ring is mapped into TC5 and TN2a beads, while the methoxycarbonyl group is mapped into an N4a bead.

**Table 1:** Property and Connectivity Matrices of Methyl 3-Furancarboxylate^a^

property matrix					
atom index	element	is in ring?	ring status	is edge?	hydrogen count
0	O	T	2	F	0
1	C	T	2	F	1
2	C	T	2	F	1
3	C	T	2	F	0
4	C	F	0	T	0
5	O	F	0	T	0
6	O	F	0	T	0
7	C	F	0	T	3
8	C	T	2	F	1

connectivity matrix									
	**0**	**1**	**2**	**3**	**4**	**5**	**6**	**7**	**8**
**0**	0	1.5	0	0	0	0	0	0	1.5
**1**	1.5	0	1.5	0	0	0	0	0	0
**2**	0	1.5	0	1.5	0	0	0	0	0
**3**	0	0	1.5	0	1	0	0	0	1.5
**4**	0	0	0	1	0	2	1	0	0
**5**	0	0	0	0	2	0	0	0	0
**6**	0	0	0	0	1	0	0	1	0
**7**	0	0	0	0	0	0	1	0	0
**8**	1.5	0	0	1.5	0	0	0	0	0

a In the property matrix, the ring status, 2, 1, 0 represents aromatic rings, nonaromatic rings, and nonring fragments, respectively. Hydrogen count values represent how many hydrogen atoms are attached to the corresponding heavy atoms. In the connectivity matrix, 0, 1.0, 1.5, and 2.0 represent no bonds, single bonds, bonds in aromatic rings, and double bonds.

**Table 2:** Mapping Array Showing Internal and External Connections and Edge Status for Methyl 3-Furancarboxylate

group index	atom index	atom index within group	outer group 1	outer atom 1	outer bonds 1	inner atom 1	inner bond 1	inner atom 2	inner bond 2
Ring Group (Group 0)									
0	0	0	–	–	–	1	1.5	4	1.5
0	1	1	–	–	–	0	1.5	2	1.5
0	2	2	–	–	–	1	1.5	3	1.5
0	3	3	1	0	1	2	1.5	4	1.5
0	8	4	–	–	–	0	1.5	3	1.5
Non-Ring Group (Group 1)									
1	4	0	0	3	1	1	2	2	1
1	5	1	–	–	–	0	2	–	–
1	6	2	–	–	–	0	1	3	1
1	7	3	–	–	–	2	1	–	–

In our framework, the tokenization process is entirely automated, enabling consistent treatment of molecules of varying architectures from polycyclic scaffolds to functionalized aromatic systems.

### 2.3 Hierarchical Mapping Strategy

Following the initial processing of the input SMILES string, the core of our framework is a hierarchical mapping strategy that translates the atomistic structure into a coarse-grained model. This procedure uses the main mapping matrix. It sorts atoms into distinct ring and nonring sections. A key principle of our approach is that bead assignment is performed dynamically. As the algorithm analyzes each section, groups of atoms that match a rule in our dictionary are immediately assigned a bead type, and this information is used to guide the mapping of subsequent, connected fragments. The algorithm is broadly divided into two main stages: the mapping of ring and nonring structures. The separation of ring (blue) and nonring (yellow) sections is illustrated in Figure 2(c). The reason behind the preference to map rings is explained below.

- **Establishing a Rigid Foundation:** Ring systems, particularly aromatic ones, are the most structurally rigid parts of a molecule. By mapping these stable foundations first, we establish a set of fixed anchor points. The more flexible nonring sections can then be mapped in the context of these rings.
- **Preventing Complications with Lone Atoms:** Many nonring sections consist of single atoms (e.g., a hydroxyl oxygen) attached to a ring. Attempting to map these lone atoms first would be problematic, as their correct bead assignment almost always depends on merging them with the larger ring structure they are attached to. By mapping the ring sections first, these lone atoms are naturally merged and assigned to a defined structure.

#### 2.3.1 Mapping Ring Structures

As mentioned before, the mapping of ring systems in coarse-grained models requires a systematic approach to preserve their structural and chemical characteristics. Because aromatic and nonaromatic rings exhibit different bonding patterns and levels of rigidity, the algorithm applies a priority-based procedure tailored to each case. We illustrate the mapping of methyl 3-furancarboxylate in Figure 2(c) and (d). The steps below outline this hierarchy, ensuring that the most critical features are captured first before completing the mapping of the full ring.

1. **Ring Fusion Points:** The algorithm first identifies and maps any atoms that are part of more than one ring system. These fusion points are the most constrained atoms in the molecule, and mapping them first provides a stable scaffold for the rest of the section. They are typically grouped with a neighbor and assigned a specialized fused-ring bead type (e.g., TC5, TC5e). We provided an example of this fusion ring in Figure 3(a) by mapping the Quinoline molecule (SMILES: C1=CC2=CC=CC=C2N=C1), where the purple bead TC5e is the fusion point.
2. **Double Bonds in Nonaromatic Ring:** Next, atoms involved in double bonds are prioritized. In aromatic rings, all atom connections share a delocalized π-bonding equivalent to alternating single/double bonds. However, double bonds can still exist in nonaromatic rings. Double bonds are more rigid than single bonds, making them the next most important structural feature to anchor. They are typically mapped as two-atom beads. If any of the atoms in the double bond are connected to a lone external section, that section is often grouped with the double-bonded atoms to form a larger, three-atom bead that captures the entire functional group.
3. **Ring Atoms with a Single-Atom Non-Ring Neighbor:** The algorithm then proceeds to map ring atoms that are connected to single-atom nonring sections. The ring atom, its unmapped ring neighbor, and the external lone atom are grouped into a three-atom bead. This step ensures small functional groups (like hydroxyls or halogens) are treated as a single chemical unit.
4. **Remaining Unmapped Atoms:** Finally, any unmapped atoms that do not belong to the previously mapped categories (ring fusion points, double bonds, or ring atoms with external substituents) are grouped to complete the ring structure. For aromatic rings, these are typically paired into two-atom beads (e.g., TC5). The resulting bead assignments for the example are shown in Figure 2(d) (TC5 and TN2a for the ring; N4a for the methoxycarbonyl side chain). For nonaromatic rings, they are often grouped into three-atom beads (e.g., SC3), a common representation in coarse-graining that effectively captures the geometry of CH_2_–CH_2_–CH_2_ fragments.

**Figure 3:**
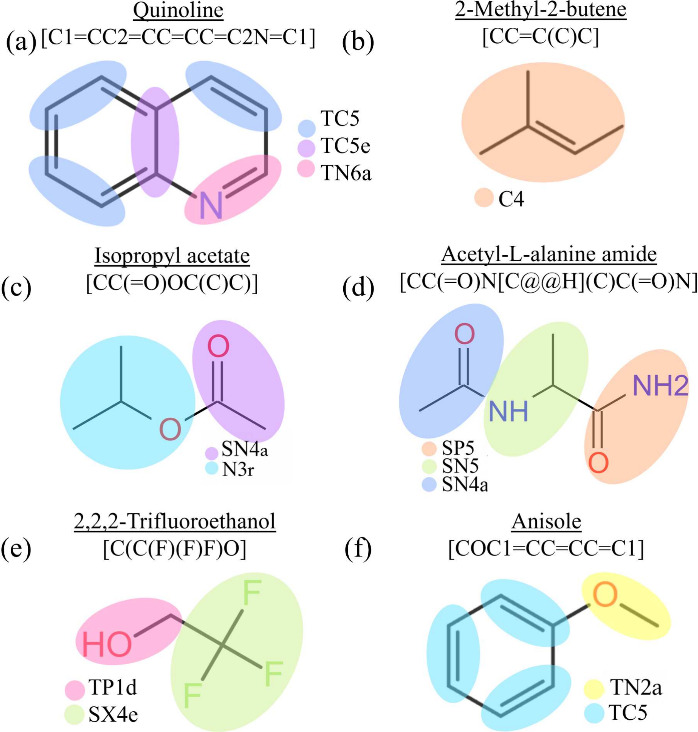
Mapping of representative molecules. (a) Quinoline is treated by the algorithm as two ring sections with a ring-fusion point, which is mapped to TC5e, while the remaining parts of the molecule are mapped to TC5 and TN6a according to their chemical structure (C=C to TC5 and C=N to TN6a). (b) 2-Methyl-2-butene, is mapped to C4, here path length, *l* is equal to 3. (c) Isopropyl acetate is treated by the algorithm as a single nonring fragment, and is mapped into two beads (SN4a and N3r), when *l* > 3. (d) Acetyl-l-alanine amide is mapped into three beads (SP5, SN5, and SN4a), for *l* > 3. (e) 2,2,2-Trifluoroethanol is mapped into two beads (TP1d, SX4e). (f) Anisole is mapped into one TN2a bead and three TC5 beads. The coloring of the beads is only tailored to each image.

#### 2.3.2 Mapping Non-Ring Structures

After all ring systems have been mapped, every remaining unmapped atom belongs to a nonring section of size at least 2 heavy atoms. The mapping of these remaining larger nonring sections is guided by the Martini 3 path length (*l*) constraint, which is defined as the maximum number of consecutive covalent bonds spanned within a single coarse-grained bead and must not exceed three (i.e., *l* ≤ 3). At this stage, all lone-atom sections have already been accounted for by being merged into the rings they were attached to.

- **Single-Bead Mappable Fragments (*l*≤ 3):** The algorithm first tests each nonring section to determine its length. If the longest path between any two atoms in the fragment is three bonds or fewer, it is mapped to a single bead (an example in Figure 3b). A canonical signature is generated based on its structure to find the correct bead type in the dictionary.
  - For linear chains, the signature is a simple string of atom and bond types (e.g., ‘CC=CC’).
  - For branched fragments, the signature is defined by the central atom followed by the signatures of each branch (e.g., ‘C­(C)­(C)­(CC)’). This ensures a unique representation regardless of atom ordering.
- **Complex Fragments Requiring Splitting (*l*> 3):** Now, if a fragment is too large, it must be broken down using a recursive splitting strategy. Here, recursive indicates that the splitting procedure is applied iteratively: the fragment is divided once, the resulting pieces are evaluated, and any sections that still violate the path length constraint are further subdivided until all fragments comply.
  - For long linear chains, the algorithm prioritizes efficiency by partitioning the chain into the largest possible mappable units, which are typically four-atom fragments (*l* = 3). For example, an octane molecule would be split into two four-carbon beads.
  - For complex branched structures, a more adaptive strategy is used. The algorithm identifies the two ‘edge’ atoms (terminals) with the shortest path between them. This path is broken off to form the first new section, and the remaining atoms form the second. This is repeated until all resulting fragments are small enough to be mapped by a single bead. In the edge case where multiple edge atoms are equidistant (e.g., in a cross-like structure like neopentane), they are grouped and broken off together. A representative example of isopropyl acetate and acetyl-l-alanine amide (see Figure 3c–e) is shown here, which requires splitting into multiple beads. Example (c) specifically shows that when that nonring section is broken apart, it forms 2 parts that are both 1-bead mappable, and one is linear and the other one is branched. Example (d) illustrates a case where a single split does not fully resolve the section into single-bead mappable fragments. After the initial division, one of the resulting fragments still exceeds the path-length limit, so the algorithm recursively applies a second split. Example (e) further illustrates the edge case where multiple edge atoms are equidistant. In this example, the three fluorine edge atoms are broken off together with their central carbon atom.
- **Resolving Functional-Group Ambiguities Using Hydrogen Counts:** In some cases, the SMILES pattern of a fragment alone is not sufficient to uniquely identify the functional group, because the same heavy-atom connectivity can correspond to multiple chemistries depending on substitution. This occurs for motifs such as amines, imines, amides, and for oxygenated groups that are ambiguous without protonation state (e.g., carboxylic acid vs ester, acetal/ketal vs hemiacetal/hemiketal, or diol-like motifs). To resolve these ambiguities without explicitly querying neighboring beads, the algorithm uses the *hydrogen counts* stored in the property matrix (see Table 3). Hydrogen counts allow us to infer how many substituent (*R*) groups are attached to a heteroatom within the fragment, which determines its substitution class and thus the correct bead assignment.
  1 **Amide/amine substitution (primary/secondary/tertiary):** for an N-centered fragment, the number of attached hydrogens distinguishes whether the nitrogen is primary (NH_2_, two H), secondary (NH, one H), or tertiary (no H). For example, the heavy-atom pattern NC­(=O) corresponds to an amide, but the N–H count differentiates a primary amide (H_2_ NC­(=O)­R), a secondary amide (HNC­(=O)­R_2_), and a tertiary amide (NC­(=O)­R_3_). In our dictionary, these are mapped to distinct Martini bead types: primary amides are assigned to **P5**, secondary amides to **P3**, and tertiary amides to **P3a**. This distinction is chemically meaningful because it changes polarity and hydrogen-bonding capability, and therefore can change the appropriate Martini bead type.
  2 **Carboxylic acid vs ester:** the fragment pattern C­(=O)­O is shared by both carboxylic acids and esters. If the oxygen in this motif carries one hydrogen (O–H), the group is a carboxylic acid (C­(=O)­OH) and is assigned to **P2**; if the oxygen has zero hydrogens, it must be substituted (C­(=O)­OR) and is therefore an ester, assigned to **N4a**. This resolves the ambiguity using only atom-local information.
  3 **Acetal/ketal vs hemiacetal/hemiketal vs diol:** acetal-like motifs contain a carbon bonded to two oxygens (e.g., C­(OR)­(OR)). The hydrogen counts on the two oxygens indicate whether either oxygen is a hydroxyl: if one oxygen has an O–H (one H) while the other is substituted (zero H), the motif is a hemiacetal/hemiketal (C­(OH)­(OR)) and is assigned to **P2**; if both oxygens have zero hydrogens, the motif is a full acetal/ketal (C­(OR)­(OR)) and is assigned to **N4a**. In the limiting case where both oxygens have O–H (each one H), the motif corresponds to a hydrate/diol-like environment (C­(OH)­(OH)), which is chemically distinct from acetals and is assigned to **P4**.
  4 **Ether vs alcohol:** oxygen-centered fragments can also be ambiguous from heavy-atom connectivity alone, because an O atom may represent either an ether oxygen (R–O–R) or a hydroxyl oxygen (R–O–H). The hydrogen count resolves this directly: an ether oxygen has zero attached hydrogens, implying two R groups, whereas an alcohol oxygen has one attached hydrogen, implying one R group. In our dictionary, ether-like environments can be assigned to **TN2a** (Figure 3f) or **N3a/r** depending on the surrounding chemical context (e.g., aromatic vs aliphatic substitution and local polarity), while alcohol-like environments can be assigned to **N6** or **P1** depending on context (e.g., phenolic vs aliphatic alcohol).

**Table 3:**
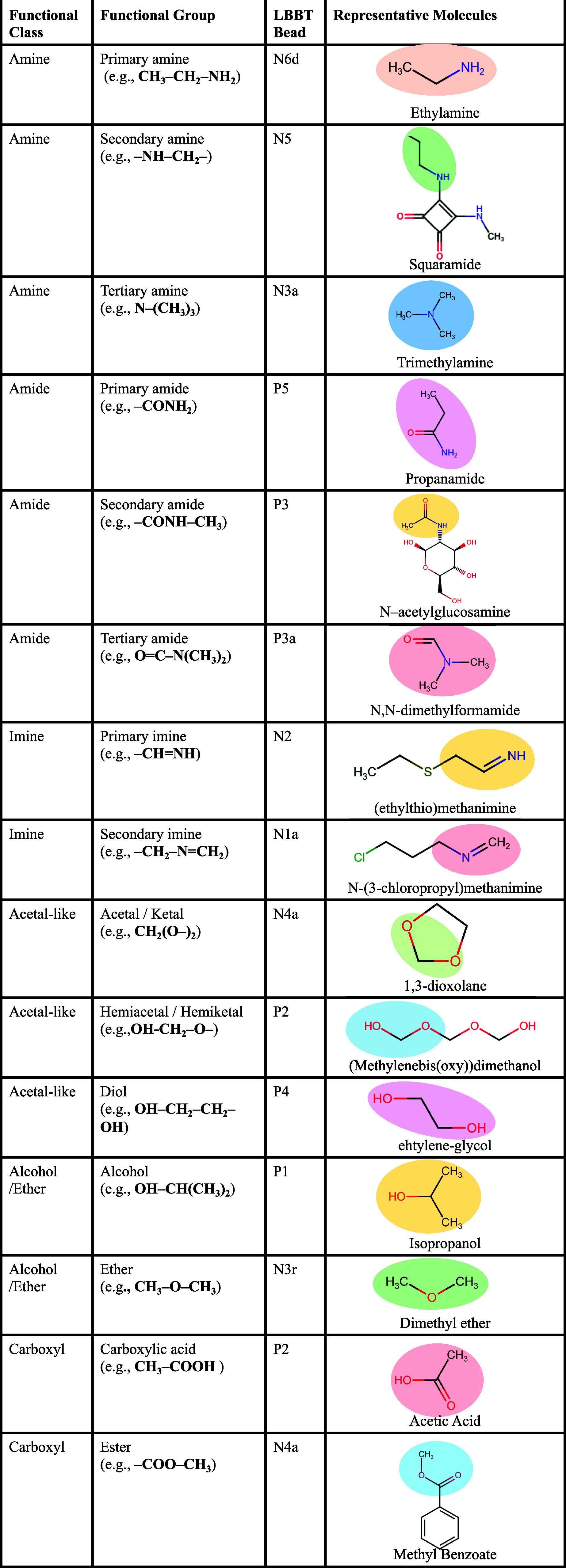
Mapping of Functional Classes and Groups to LBBT Beads^a^

a The table shows bead type reassignment; bead size resolution (R, S, T) follows standard Martini 3 definitions. The building blocks of alcohol, ether, carboxylic acid, and ester are mapped to Martini 3 beads, identical to those from Martini 3 datasets.^39,41^ The amine and imine groups are mapped to the beads, same as reported by the Grünewald dataset.^67^.

### 2.4 Output Generation

After this final step, every atom in the original molecule has been assigned to a coarse-grained bead, and the mapping is ready for output generation. It is essential to develop an algorithm that not only performs bead mapping but also generates bead coordinates and bond lengths, thereby producing a consistent coordinate set and bond topology. To begin this step, the initial three-dimensional coordinates of the all-atom (AA) structure are obtained using RDKit,^68^ which generates a conformer from the input SMILES string. Once a molecule is generated from its SMILES string and mapped to beads, each bead corresponds to a specific set of atoms. The center of geometry of these atoms is then computed to define the bead’s coordinate. This ensures bead positions correspond to the AA geometry. The bond length between two connected beads is then obtained as the Euclidean distance between their respective centers of geometry.

### 2.5 Bond and Angle Potential Parameters from xTB-Based Ensemble Sampling

Bonded parameters are derived from constant temperature sampling of an AA reference trajectory generated using an extended tight-binding workflow,^52^ followed by the approach taken in Bartender.^51^ The objective is to obtain equilibrium bond lengths and angles, as well as their corresponding force constants, from statistically meaningful fluctuations rather than from a single conformer. Starting from a SMILES representation, a three-dimensional structure is generated using RDKit^69^ with ETKDG^70^ embedding, followed by the Universal Force Field optimization.^71^ This structure is subsequently optimized using the GFN-FF Hamiltonian within the xTB framework. GFN-FF is selected because it provides computational efficiency and stable sampling suitable for high-throughput mapping workflows, while maintaining chemically consistent geometries. Following optimization, a finite-temperature classical molecular dynamics simulation is performed using xTB at *T* = 300 K in the NVT ensemble. This produces 1000 frames sampling conformational fluctuations around the optimized structure. The simulation parameters are

- Temperature: 300 K
- Total simulation time: 10 ps
- Integration time step: 0.1 fs
- Trajectory output interval: 10 fs

Each trajectory frame is mapped to a CG representation using the COG of the atoms assigned to each bead, including all explicitly added hydrogens. The use of COG ensures consistency with the Martini mapping criterion adopted in Martini 3 and avoids mass-weighting biases that would otherwise alter fluctuation statistics. The details of the bond and angle equilibrium values and their corresponding force constant are detailed in Section S1 of the Supporting Information (SI).

### 2.6 Ability to Map Molecules with >170 Heavy Atoms

To evaluate the capability of the developed framework, we examined the size distribution of molecules that could be automatically mapped. Specifically, we assessed how well the algorithm performs for molecules containing different numbers of heavy atoms. Heavy atoms refer to all non-hydrogen atoms (e.g., C, N, O, S, halogens). For this analysis, we used^42,67,72–75^ representative data sets of increasing chemical diversity and size: the Bereau data set,^42^ the Grünewald data set,^67^ the Kaggle log *P* data set,^72^ and the 2D log *P* Molecular Benchmark.^73,74^ To further probe the scalability of the algorithm, we also analyzed the TPCN (Terpenoids Content Network) database.^75^ The TPCN is a curated collection of over six thousand naturally occurring terpenoids derived from 1254 plant species across 156 families.

As shown in Figure 4, our algorithm can successfully map not only the smaller organic molecules from the Bereau, Kaggle, 2D, and Grünewald data sets, typically containing up to 16 heavy atoms, but also the larger, more intricate molecules from the TPCN data set, which span a broad range of sizes and include species exceeding 170 heavy atoms. This demonstrates the robustness and transferability of the framework for large-scale molecular applications. The accuracy of the coarse-grained models of these molecules has been evaluated in Section 4.

**Figure 4:**
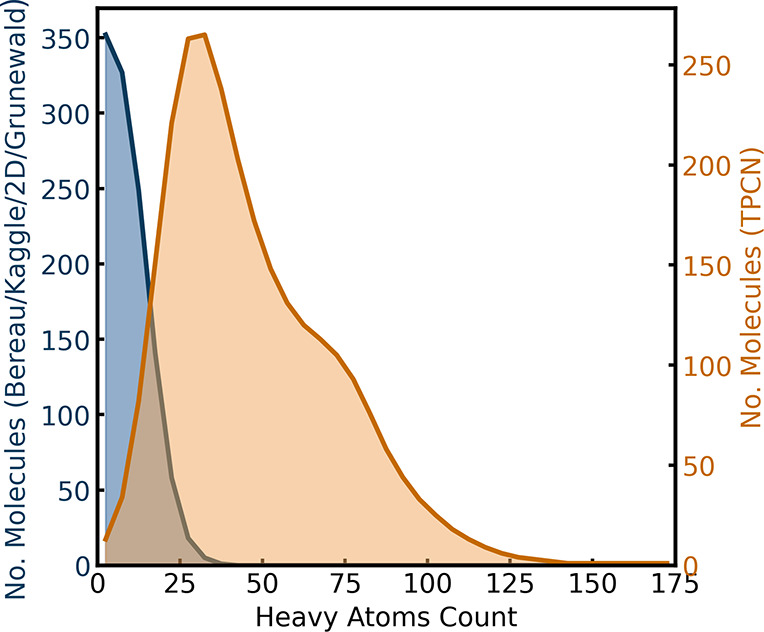
Distribution of successfully mapped molecules with varying heavy-atom counts is shown in blue for all four data sets: Bereau,^42^ Kaggle,^72^ 2D,^73^ and Grünewald^67^ data set, and in orange separately for the TPCN data set.^75^

## 3 Simulation Details

To assess the reliability and physical consistency of the generated coarse-grained models, we performed two levels of validation: (i) thermodynamic integration (TI) calculations for transfer free energies, and (ii) equilibrium stability simulations in the NPT ensemble (see results in Section 4.4. In this work, we used the TI^76^ to compute the free energy from water, hexadecane, chloroform, and octanol using Martini 3 force field parameters. For each successfully mapped molecule, the pipeline-generated coordinate (.gro) and topology (.itp) files were directly incorporated into GROMACS for TI calculations in four separate simulation environments: pure water, hexadecane, chloroform, and hydrated 1-octanol with 0.2 M of water. Within the Martini 3 framework, these species are modeled using the W bead, a four-bead C1 chain, an SC2–SC2–SP1 representation, and the X2 bead type, as detailed in ref.^39^ To reproduce experimentally relevant conditions, the octanol phase was modeled in its water-saturated form by incorporating water at a mole fraction of 0.2.^77^ Simulations employed the stochastic dynamics (sd) integrator with a time step of 20 fs (dt = 0.020 ps). Each λ-window was simulated for 4 ns, with 20 intermediate λ values for van der Waals decoupling (0.0 → 1.0) and simultaneous electrostatic decoupling, defined as λ = 0, 0.05, 0.1, 0.15, 0.2, 0.25, 0.3, 0.35, 0.4, 0.45, 0.5, 0.6, 0.65, 0.7, 0.75, 0.8, 0.85, 0.9, 0.95, 1.

Soft-core potentials were applied with parameters sc-power = 1, sc-alpha = 0.5, and sc-r-power = 6 to ensure smooth decoupling and avoid singularities. All simulations used the Verlet cutoff scheme with *r*_vdw_ = *r*_coul_ = 1.1 nm, a shifted potential for both van der Waals and Coulomb interactions (Potential-shift-verlet). The temperature and pressure of the simulation have been set to 300 K using the v-rescale thermostat (τ_*t*_ = 1.0 ps) and at 1.0 bar using the Parrinello–Rahman barostat (τ_*p*_ = 2.0 ps, compressibility = 4.5 × 10^–5^ bar^–1^). Free energy differences Δ*G* between the coupled and decoupled states for both water and octanol bath were computed separately from the TI output. From their free energy differences, we computed log *P* as

$$\log P=\frac{\Delta G_{\mathrm{OW}}}{2.303RT}$$ 1

For water/hexadecane, and water/chloroform, we simultaneously obtained the respective difference in free energy. To ensure the robustness of our thermodynamic integration protocol, we first benchmarked the methodology against the reference Martini 3 bead hydration and solvation free energies reported in the SI of Souza et al.^39^ (see Figure S1 of Section S2 in the Supporting Information). For each bead type, free energies of transfer in both water and octanol were reproduced within statistical uncertainty, yielding a near-perfect linear correlation with published values (*R* ^2^ = 0.99). This validation step confirms that the present TI setup faithfully reproduces the Martini 3 reference data before extending it to small-molecule systems.

For the equilibrium stability simulations, the integration time step was set to 20 fs and ran for 10 ns. If any molecule failed to complete 10 ns simulations, the respective one has been further simulated with 10 and 5 fs or lower time step. The systems were maintained at 300 K temperature and 1.0 bar pressure with the same thermostat and barostat parameters as the TI simulation.

## 4 Results and Performance Evaluation

In this section, we discuss the performance of our framework. We first validated our algorithm on mapping the original 90 molecule data set and then test its ability on chemically diverse and publicly available four molecular databases (the Bereau data set, the Kaggle log *P* data set, the 2D log *P*, and TPCN Molecular database) by generating valid Martini 3 mappings. Among them, we computed Δ*G* or log *P* values and benchmarked them against experimental references for the first three data sets due to affordable computational cost. These comprise a chemically diverse library of natural and synthetic compounds enriched in intricate topologies, including fused and overlapping ring systems, polycyclic scaffolds, and rings of uncommon sizes. It provide an ideal stress for a rule-based mapping procedure, ensuring that the framework can accommodate challenging chemistries encountered in real-world applications.

The complete workflow of our framework was evaluated as a three-stage process:

1. Generation of GROMACS-specific .itp and .gro. The .itp file contains bead types, bond, and angle information. The .gro file contains the bead coordinates.
2. Successful passage through GROMACS MD Engine.
3. Completion of TI simulations in water and hydrophobic solvents (hexadecane/chloroform/hydrated octanol), and give Δ*G* difference between water and hydrophobic solvent as output.

We consider a successful mapping only when it can pass through all these three steps, and is termed “working”. In total, our framework successfully generated coarse-grained models of 6280 molecules (“Working”) over 8850 molecules. The remaining 2570 molecules (“Not Working”) failed primarily due to chemical fragments that were not yet represented in the mapping dictionary, rather than breakdowns in mapping logic or simulation stability.

### 4.1 Validation on the Martini 3 Small-Molecule Data Set

Following the protocol of Martini 3 parametrization, we performed here the transfer free energy of three solvent pairs for the Original 90 molecule data set from the Martini 3 small molecule data set.^41^ A mapping figure of side-by-side comparison of mapped molecules generated by Martini_Mapper against the human-made model^41^ is shown for a set of molecules in Figure S2 of Section S3 in the Supporting Information. The three solvent pairs are the following: water/hydrated octanol (0.2 M water), water/hexadecane, and water/chloroform. The details of the simulations are given in the Section 3. As mentioned, all transfer free energies were computed using a consistent TI protocol. The integration time step for the TI simulations was set to 20 or 10 fs, depending on whether the molecule successfully completed the equilibrium stability simulation at the corresponding time step. The resultant free energy for a given molecule for each of the solvent pairs is plotted in *x*-axis against the experimental data for that molecule on the *y*-axis. The experimental data for all the molecules are taken from refs 78–83 . We show the correlation between calculated and experimental transfer free energies in Figure 5.

**Figure 5:**
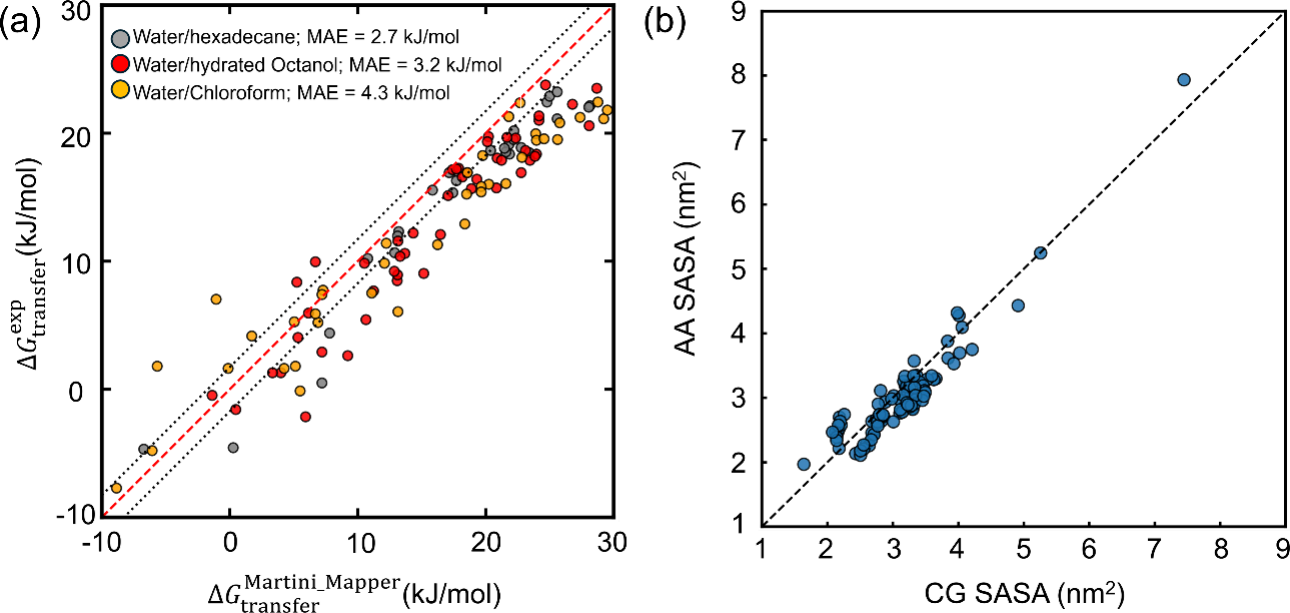
Thermodynamic and structural validation of Martini_Mapper on the complete 90-molecule Martini 3 small-molecule data set. (a) Correlation between calculated and experimental transfer free energies (Δ*G*_transfer_) for three solvent pairs: water/hexadecane (gray), water/hydrated octanol (0.2 M water, red), and water/chloroform (yellow). The central dashed line represents ideal agreement (*y* = *x*), and the dotted lines indicate a ±2.5 kJ mol^–1^ deviation from the identity line, corresponding to the commonly adopted Martini accuracy threshold. The coefficients of the three types of transfer free energies: water/hexadecane, water/hydrated octanol, and water/chloroform, are 0.82, 0.71, and 0.59, respectively. Experimental data are obtained from refs 78–83 . (b) Comparison of SASA between CG models generated by Martini_Mapper and their corresponding AA reference structures from ref 41. Each point represents one molecule. The dashed line indicates perfect agreement (*y* = *x*). The resulting correlation is *R* ^2^ = 0.877 with RMSE = 0.293 nm^2^, demonstrating preservation of molecular size and surface exposure in the automated mapping workflow.

From Figure 5a, we can see that the correlation yields *R* ^2^ value of 0.82 for water/hexadecane, 0.71 for water/hydrated octanol, and 0.59 for water/chloroform. To further obtain the deviation from the given experimental data set, we computed the mean absolute error (MAE), which came as 2.7 kJ/mol for water/hexadecane compared to 3.09 kJ/mol obtained by Auto-MartiniM3; 3.2 kJ/mol for water/hydrated octanol compared to 2.33 kJ/mol by Auto-MartiniM3 and 4.3 kJ/mol for water/chloroform compared to 3.57 kJ/mol by Auto-MartiniM3. It is to be noted that the MAE obtained from Martini_Mapper is higher than 2.5 kJ/mol, the threshold value adopted in Martini parametrization;^41^ however, the average deviation across all the solvent pairs is not significantly differ than what is obtained using Auto-MartiniM3. The deviation in Martini_Mapper is attributed to the absence of virtual site, dihedral, and improper dihedral terms.

In addition to thermodynamic validation, we assessed structural consistency by comparing SASA of the CG models with their corresponding AA reference structures. SASA values were computed using the GROMACS gmx sasa utility with a probe radius of 0.14 nm for AA models and 0.191 nm for CG models, consistent with Martini conventions.^41^ The details of the SASA calculation are detailed in Section S4 of the Supporting Information. As shown in Figure 5b, the CG and AA SASA values exhibit strong correlation, with *R* ^2^ = 0.877 and RMSE = 0.293 nm^2^. The CG models show a slight systematic underestimation of SASA, which can be attributed to reduced rigidity in the coarse-grained representation relative to atomistic structures. Nevertheless, the high correlation demonstrates preservation of molecular size and surface exposure across chemically diverse molecules.

All these results confirm that Martini_Mapper successfully reproduces the Martini 3 small-molecule data set in a fully automated manner. The workflow generates simulation-ready topologies for all 90 molecules, preserves molecular size and surface characteristics, and reproduces global partitioning trends across multiple solvent environments. While the quantitative accuracy does not yet match manually optimized parametrizations, the framework provides a transparent and reproducible baseline coarse-grained representation that can serve as a foundation for subsequent targeted refinement.

### 4.2 Validation on Independent Partitioning Data Sets

To evaluate the predictive reliability of Martini_Mapper as a fully automated coarse-graining framework, we assessed its performance on three independent data sets of experimentally measured water–octanol partitioning thermodynamics. All molecules were mapped using the LBBT dictionary in a single-pass automated workflow, and transfer free energies were computed using a consistent simulation protocol across data sets. It is to note that only molecules that successfully completed 10 ns NPT stability simulations in pure water (as described in Section 3) were subsequently subjected to TI calculations using the 20 fs time step. All the itp/gro files of these data sets are available in GitHub: https://github.com/eliobaby/Martini_Mapper/tree/main/Working_Molecules.

#### Bereau Data Set

The TI simulation results for the Bereau data set include 427 molecules that were successfully completed under NPT conditions. This data set consists of structurally diverse neutral organic compounds with experimentally measured water–octanol transfer free energies. Figure 6a shows the correlation between calculated and experimental Δ*G*_OW_ values for the working subset. The resulting correlation yields *R* ^2^ = 0.68, RMSE = 5.69 kJ mol^–1^, and MAE = 4.22 kJ mol^–1^. While systematic deviations remain, the overall trend in hydrophobicity is captured across a broad chemical space without molecule-specific tuning. These results indicate that Martini_Mapper provides a consistent baseline description of partitioning thermodynamics, suitable as an initial automated model that can subsequently be refined when higher quantitative accuracy is required.

**Figure 6:**
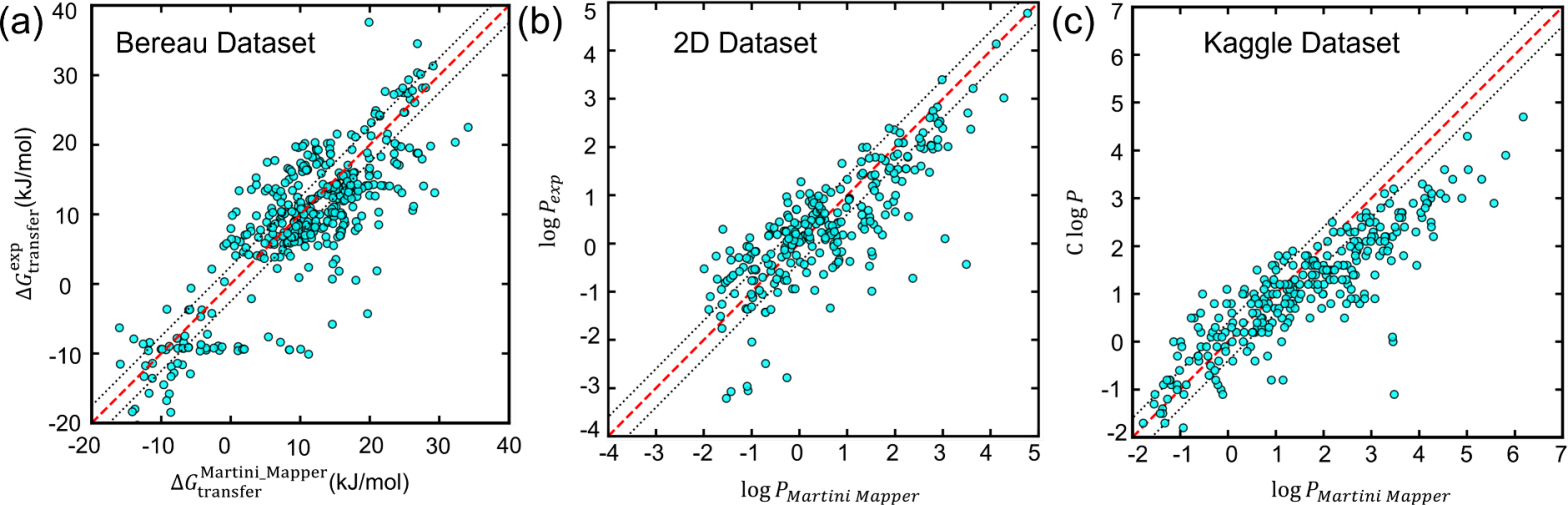
Validation of Martini_Mapper on three independent partitioning data sets: (a) Bereau, (b) 2D data set, and (c) Kaggle data set. The dashed line represents ideal agreement (*y* = *x*), and the dotted lines indicate a ±2.5 kJ mol^–1^ (±0.48 log *P*) deviation from the identity line, corresponding to the commonly adopted Martini accuracy threshold.^41^ Δ*G*_OW_ for the Bereau data set is the transfer free energies from water to hydrated octanol (0.2 M water). For (a) the Bereau data set, the resulting correlation yields *R* ^2^ = 0.68, RMSE = 5.69 kJ mol^–1^, and MAE = 4.22 kJ mol^–1^. For (b) the 2D data set, the resulting metrics are *R* ^2^ = 0.52, RMSE = 0.838, and MAE = 0.632 in log *P* units. For (c) the Kaggle data set, the resulting performance metrics are *R* ^2^ = 0.40, RMSE = 0.95, and MAE = 0.72 in log *P* units.

#### 2D Benchmark Data Set

We next evaluated generalizability using the independent 2D molecular benchmark data set. In this data set, we ran TI of 267 molecules, which were successfully run with 20 fs NPT production run for 10 ns. As shown in Figure 6b, the correlation between predicted and experimental log *P* values yields *R* ^2^ = 0.52, RMSE = 0.838, and MAE = 0.632 in log *P* units. The preservation of moderate correlation on an unseen data set indicates that the workflow captures global physicochemical trends despite the absence of data set-specific refinement.

#### Kaggle Data Set

Finally, we examined performance on the Kaggle log *P* data set, which contains chemically diverse small molecules. In this data set, we ran TI of 291 molecules, which were successfully run with 20 fs NPT production run for 10 ns. The resulting correlation (Figure 6c) yields *R* ^2^ = 0.40, RMSE = 0.95, and MAE = 0.72 in log *P* units. The reduced correlation relative to the Bereau and 2D data sets is reasonable with broader chemical diversity and fragment coverage limitations of the current dictionary, which affect the quantitative fidelity of the first-pass CG representation.

Across all three independent data sets, the results demonstrate that Martini_Mapper provides a consistent and fully automated baseline coarse-grained representation without molecule-specific optimization or iterative tuning. The observed quantitative deviations therefore reflect the combined effects of dictionary coverage, bead assignment choices, bonded-term simplifications, and the absence of data set-specific refinement. Rather than attributing these deviations to intrinsic limitations of coarse-graining, the present benchmarks define the current performance envelope of the automated workflow under a fixed and reproducible protocol.

### 4.3 Structural Validation of Larger Molecules

To evaluate the structural consistency of coarse-grained models for larger systems, we performed a quantitative comparison of SASA between Martini_Mapper-generated CG models and their corresponding AA reference structures of molecules from the TPCN data set. The AA structures were obtained from our xTB-based optimization pipeline, ensuring internal methodological consistency. Due to the large number of constraints present in highly rigid systems and the current absence of automated virtual-site generation, full thermodynamic validation via transfer free energy calculations was not performed for the entire TPCN data set. Instead, SASA was used as a structural validation metric, consistent with established Martini 3 parametrization principles where preservation of molecular volume and surface characteristics is essential. A representative subset of 560 molecules from the TPCN data set was analyzed, spanning systems containing 9 to 75 heavy atoms. The path to this data set is GitHub: https://github.com/eliobaby/Martini_Mapper/tree/main/Working_Molecules/TPCN/xtb_2. As shown in Figure 7, CG and AA SASA values exhibit excellent agreement with a correlation coefficient of *R* ^2^ = 0.960 and RMSE = 0.401 nm^2^. The strong linear correlation indicates that the mapping procedure preserves overall molecular size and surface properties even for larger and more complex systems. It is to be noted that the full set of 4716 molecules mapped using Martini_Mapper was not included in the SASA comparison to reduce computational cost. This exclusion was solely due to resource considerations and does not indicate any limitations of the Martini_Mapper.

**Figure 7:**
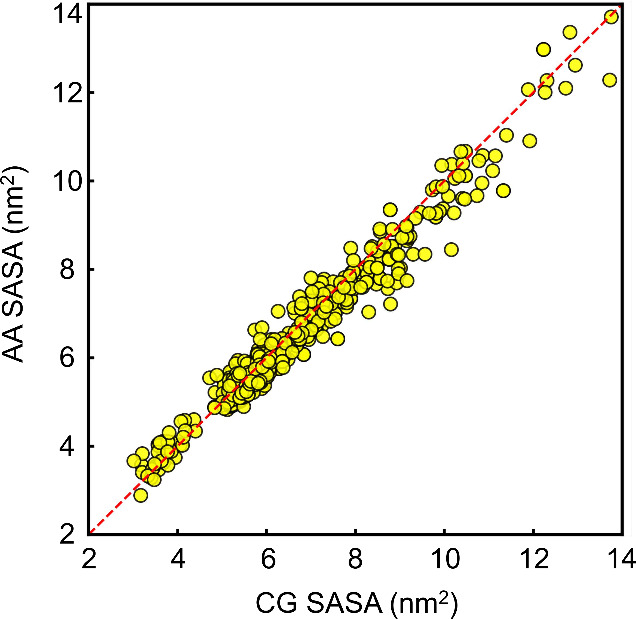
Comparison of SASA for 560 TPCN molecules between coarse-grained models generated by Martini_Mapper and corresponding all-atom reference structures obtained from the xTB pipeline. Each point represents one molecule. The dashed red line indicates ideal agreement (*y* = *x*). The correlation between CG and AA SASA values is *R* ^2^ = 0.960 with RMSE = 0.401 nm^2^, demonstrating strong preservation of molecular volume and surface characteristics across systems containing 9–75 heavy atoms.

### 4.4 Computational Performance and Numerical Stability

In addition to thermodynamic and structural validation, we evaluated the computational efficiency and scalability of the Martini_Mapper workflow. The core mapping time as a function of molecular size (number of heavy atoms) is presented in Figure 8. The reported timings correspond exclusively to the rule-based mapping stage and explicitly exclude the xTB-based coordinate generation and bonded parameter refinement step (i.e., executed using the –no–xtb flag). Each molecule was mapped using a single CPU core (Intel Xeon E5–2680v2 processor). The results demonstrate near-linear scaling of mapping time with molecular size across a broad range of compounds, indicating that the algorithm maintains computational tractability even for larger fragments.

**Figure 8:**
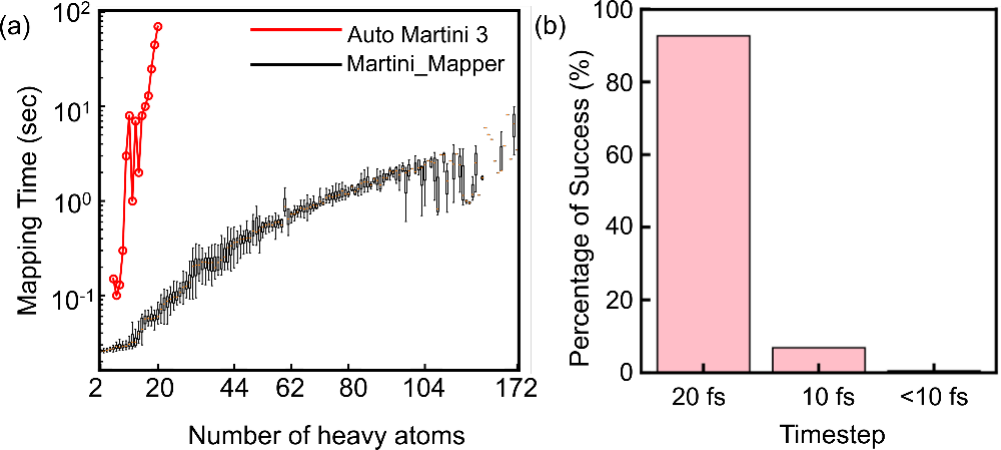
Performance and stability analysis of the Martini_Mapper workflow. (a) Core mapping time as a function of molecular size (number of heavy atoms). Each data point corresponds to a single molecule mapped using one CPU core (Intel Xeon E5–2680v2 processor). The data set includes molecules from the original 90-molecule benchmark, Bereau set, 2D data set, Kaggle data set, Grünewald set, and the TPCN database. Black symbols represent Martini_Mapper timings obtained without the bond/angle potential parameter refinement stage, while red symbols indicate mapping times obtained using Auto-MartiniM3.^45^ The near-linear trend on the log–log scale demonstrates scalable performance of the Martini_Mapper framework. (b) Percentage of successful coarse-grained molecular dynamics simulations as a function of integration time step. The majority (>90%) of automatically generated models remain stable at the standard Martini time step of 20 fs, indicating that the automatically generated bonded parameters yield numerically stable models for standard Martini simulation settings. In testing the success percentage, all the simulations were performed in NPT ensembles with a production runtime of 10 ns.

For example, a molecule containing 20 heavy atoms requires approximately 0.07 s using Martini_Mapper, whereas Auto-MartiniM3 ^45^ requires approximately 70 s under comparable single-molecule conditions. This difference arises from the fully deterministic and fragment-based nature of the Martini_Mapper algorithm. The observed scaling behavior confirms that the computational complexity is dominated by graph traversal and fragment assignment operations, which scale approximately linearly with molecular size. Consequently, the automated mapping procedure achieves substantially lower wall-time per molecule while remaining systematic and reproducible. These timings correspond exclusively to the core fragment-based mapping stage. The difference reflects the deterministic fragment-based mapping strategy employed in Martini_Mapper.

We further assessed the numerical robustness of the generated coarse-grained models by performing CG molecular dynamics simulations using different integration timesteps. The percentage of successful simulations as a function of time step is also shown in Figure 8. The majority of automatically generated models remain stable at the standard Martini time step of 20 fs, demonstrating that the bonded parameters and constraint treatment produce numerically stable systems suitable for routine CG-MD simulations. These results confirm that the workflow combines computational efficiency with physically consistent model generation. These benchmarks provide a comprehensive evaluation framework encompassing thermodynamic accuracy, structural consistency, computational efficiency, and simulation robustness, enabling direct comparison of Martini_Mapper with existing automated and manually curated Martini 3 parametrization approaches.^41,45^

## 5 Limitations of Martini_Mapper

While the automated framework substantially advances the process of generating Martini 3 coarse-grained models, several important limitations remain. The first limitation we want to discuss is related to our bead dictionary. As a result, the current ruleset is most reliable for molecules dominated by carbon (C), oxygen (O), and nitrogen (N), and less robust when encountering sulfur-, phosphorus-, or halogen-containing fragments, or other motifs not present in the original data set. Therefore, it is fundamentally more successful in mapping, which is tied to the coverage of its bead dictionary.

The mapping procedure itself introduces algorithmic constraints. The strict requirement that path lengths within a bead remain three bonds or fewer ensures physical plausibility, but it can also exclude chemically reasonable mappings for highly branched structures or unusual topologies. Enforcing molecular symmetry can fail when stereocenters or asymmetric substituents are present, since chirality is not explicitly represented. Recursive splitting of large fragments may yield multiple mathematically valid partitions, but the current algorithm lacks a mechanism to rank these alternatives by chemical realism. These features reflect the deterministic and rule-based nature of the present implementation.

The present implementation performs a single-pass bead assignment without iterative optimization against multiple thermodynamic targets. Bead types are selected deterministically from the dictionary and are not refined through feedback from experimental observables. As a result, the reported accuracy represents a reproducible first-pass parametrization rather than a fully optimized Martini 3 model. Furthermore, though the current workflow automatically generates bond and angle parameters, it does not generate proper or improper dihedral terms. Planarity in rigid or fused aromatic systems is therefore maintained primarily through the angle network and constraints. Automated generation of dihedral and improper potentials remains a limitation of the present framework. Similarly, virtual-site construction is not implemented. Manually curated Martini models often introduce virtual sites to preserve rigidity and improve numerical stability in planar systems. The absence of automated virtual-site generation may limit structural fidelity in certain highly rigid molecules. Very stiff bonded interactions are treated thus as constraints to maintain numerical stability at standard Martini timesteps. While this approach ensures robust simulations, it may restrict fine control over highly rigid or torsionally complex systems.

Finally, validation across independent data sets reflects the current performance envelope of the automated workflow under a fixed and reproducible protocol. The observed deviations across chemically diverse data sets therefore reflect the combined effects of dictionary coverage, deterministic bead assignment, bonded-term simplifications, and the absence of data set-specific refinement. Future development will focus on expanding dictionary coverage, incorporating automated dihedral and virtual-site generation, and introducing iterative optimization strategies to improve transferability while preserving automation.

## 6 Conclusion

In this work, we introduced a fully automated framework that transforms SMILES strings of a molecule into Martini 3 coarse-grained models through a systematic, rule-based algorithm. By formalizing the mapping procedure into a dictionary-driven workflow, the approach reduces subjective variability in bead assignment and reliance on chemical intuition, thereby addressing a practical bottleneck in high-throughput coarse-grained model construction. The framework was evaluated on chemically diverse data sets and benchmarked against experimental log *P* and transfer free energy values across multiple solvent systems. The benchmarking results demonstrate that automated mapping can produce reproducible, simulation-ready Martini 3 topologies across molecules ranging from small systems to compounds up to 172 heavy atoms, with quantitative performance comparable to existing automated approaches. In total, our framework successfully mapped 6280 molecules across six data sets: 542 from Bereau, 300 from 2D, 332 from Grünewald, 300 from Kaggle, 4716 from TPCN, and 90 from the Original 90 molecule data set.

The significance of this advance lies in making coarse-grained simulation more accessible, reproducible, and scalable. The primary challenge in small-molecule parametrization is not the absence of standards, but the consistent application of detailed guidelines in high-throughput contexts. Martini_Mapper is designed to automate and scale the systematic application of established Martini 3 conventions. By replacing manual mapping with an automated and extensible pipeline, the framework enables systematic treatment of large chemical libraries while maintaining consistency across molecules. In direct comparison with recent automated methodologies such as AutoMartini3, the present implementation achieves thermodynamic accuracy comparable to recent automated approaches, while substantially reducing computational overhead during the core mapping stage.

While the current framework establishes a robust foundation, several clear avenues remain for future development. The most immediate priority is the systematic expansion of the bead dictionary. Although the present rules capture a broad range of carbon-, oxygen-, and nitrogen-containing chemistries, coverage of sulfur, phosphorus, halogens, and metal coordination environments remains incomplete. Targeted inclusion of fragments from chemically diverse libraries will progressively reduce mapping failures and improve coverage across broader chemical space. A second direction concerns refinement of bonded and nonbonded parameter treatment. The present workflow performs deterministic bead assignment without iterative refinement against multiple thermodynamic targets. Systematic expansion of the benchmarking data set, and incorporation of automated dihedral and virtual-site generation would improve transferability and structural fidelity. Validation against observables beyond log *P* and Δ*G*_OW_ may further strengthen robustness across chemically diverse systems.

In conclusion, these results present the current implementation of Martin_Mapper as a reproducible and scalable baseline for automated Martini 3 parametrization. Continued development will focus on expanding chemical coverage, incorporating higher-order bonded terms, and improving transferability through systematic validation, while maintaining the deterministic and high-throughput character of the workflow.

## Supplementary Material



## Data Availability

The data underlying this study, including all the gro/itp files of the working molecules and open source codes for Martini Mapper, are openly available at https://github.com/eliobaby/Martini_mapper.

## Notes

Additional materials that complement the main manuscript. **Section S1** presents the formal derivation of bond and angle equilibrium values and corresponding force constants. **Section S2** and **Figure S1** report hydration free-energy benchmarks for 106 Martini 3 beads, comparing thermodynamic integration results obtained in this work with reference Martini 3 values. **Section S3** and **Figure S2** compares representative small-molecule mappings generated by Martini_Mapper with expert-curated Martini 3 models.**Section S4** details the SASA calculation protocol for both atomistic and coarse-grained models (PDF)
