# Supplementary material for: Martini Mapper: An Automated Fragment-Based Mapping Algorithm for Developing Coarse-Grained Models within the Martini 3 Framework
Source: J Chem Inf Model. 2026 Apr 28;66(9):5272–86. doi: 10.1021/acs.jcim.5c02903 (PMC13169355; doi:10.1021/acs.jcim.5c02903)
Supplement: Supplementary file 1 [file ci5c02903_si_001.pdf]

Supporting Information: Martini Mapper: An Automated Fragment-Based Mapping Algorithm for Developing Coarse-Grained Models within the Martini 3 Framework

Kevin V. Bigting,[†,¶] Shubhadeep Nag,[‡,¶] and Yaxin An[*,‡]

†*Division of Computer Science and Engineering, Louisiana State University, Baton Rouge, LA, 70803*

‡*Department of Chemical Engineering, Louisiana State University, Baton Rouge, LA, 70803*

¶*K.V.B. and S.N. contributed equally to this work.*

E-mail: yxan@lsu.edu

S1 Bond and Angle Equilibrium Values and Force Constant

Bond equilibrium values and force constant: For each bonded bead pair (i, j), the bond distance $r_{ij}(t)$ is computed for every trajectory frame. The equilibrium bond length is defined as the ensemble mean:

$$r_{ij}^0 = \langle r_{ij} \rangle \tag{S1}$$

where $\langle \cdot \rangle$ denotes averaging over all sampled frames. Assuming harmonic fluctuations consistent with the Martini bonded potential,

$$U_{\mathrm{bond}}(r) = \frac{1}{2} k_{ij} (r - r_{ij}^0)^2 \tag{S2}$$

the fluctuation–dissipation relation gives:

$$\langle (r - r_{ij}^0)^2 \rangle = \frac{k_B T}{k_{ij}}. \tag{S3}$$

Thus, the bond force constant is obtained directly from the variance:

$$k_{ij} = \frac{k_B T}{\sigma_{ij}^2}. \tag{S4}$$

Here, k_B is the Boltzmann constant expressed in kJ mol^{-1} K^{-1}, and $T = 300$ K. Bond distances are expressed in nm, resulting in force constants in kJ mol^{-1} nm^{-2}, consistent with Martini 3 conventions. If k_{ij} exceeds 2×10^4 kJ mol^{-1} nm^{-2}, the interaction is converted to a constraint, following the criterion adopted in Bartender.[1]

Angle equilibrium values and force constant: For each angle triplet (i, j, k), the instantaneous angle $\theta_{ijk}(t)$ is computed in radians. Because angular variables are periodic,

equilibrium values are obtained using circular statistics:

$$\theta^0_{ijk} = \mathrm{atan2}\left(\langle \sin\theta \rangle, \langle \cos\theta \rangle\right) \tag{S5}$$

Using the harmonic angle potential form:

$$U_{\mathrm{angle}}(\theta) = \frac{1}{2}k_\theta(\theta - \theta^0_{ijk})^2, \tag{S6}$$

The corresponding fluctuation–dissipation relation gives:

$$k_\theta = \frac{k_B T}{\sigma^2_\theta}. \tag{S7}$$

Angles are internally treated in radians, yielding force constants in kJ mol^{-1} rad^{-2}, consistent with Martini functional forms. The parameter extraction assumes that local fluctuations are effectively harmonic around a single minimum. This is consistent with the bonded potential form employed in Martini 3. Multi-well or strongly anharmonic behavior is not explicitly treated; instead, the resulting parameters represent effective harmonic descriptions of the sampled ensemble. At present, the automated extraction is limited to bond and angle terms. Dihedral and improper parameters are not generated within this workflow and instead follow the standard Martini bonded definitions when required.

S2 Validation of the Thermodynamic Integration Protocol

In this section, we validate our thermodynamic integration (TI) protocol by reproducing the reference hydration free energies of Martini 3 beads reported by Souza et al.[2] A total of 106 bead types were simulated individually in water using the same TI setup described in Section 3 of the main manuscript (see Figure S1. Free energy differences between the coupled and decoupled states were computed for each bead and compared directly with the

published reference data. This benchmark ensures that the present simulation parameters and TI implementation accurately reproduce the Martini 3 reference thermodynamics prior to application to small-molecule systems.

S3 Visual Comparison with Human-Curated Martini 3 Models

To further assess the accuracy of the automated mapping procedure, we provide a direct visual comparison between Martini_Mapper-generated models and the manually curated Martini 3 small-molecule models reported in Ref.[3] Figure S2 presents side-by-side representations for four representative molecules, highlighting bead types, placement, and connectivity. Differences in bead types are indicated in red. Three molecules (1-methylimidazole, 4-bromoanisole, and 1,2-dichlorobenzene) are mapped identically in terms of bead assignment and connectivity, while caffeine differs in three bead types. For the broader Original 90 dataset, most non-identical cases involve only one or two bead-type substitutions and/or the absence of manually introduced virtual sites used to enforce planarity in curated models.

S4 Solvent Accessible Surface Area (SASA) Calculation

To assess structural consistency between AA and CG models, we computed the SASA using the `gmx sasa` utility in GROMACS. For atomistic reference structures, a probe radius of 0.14 nm was used.[4] For Martini 3 CG models, a probe radius of 0.191 nm was adopted, consistent with the T-bead size in the Martini framework.[2] In Martini 3, the effective vdW radii of R-, S-, and T-beads are 0.264, 0.230, and 0.191 nm, respectively. These radii correspond to the position of the minimum of the Lennard–Jones potential, defined as

$$r_{\mathrm{vdW}} = r_{\mathrm{min}} = \frac{d_{\mathrm{min}}}{2} = \frac{\sqrt[6]{2}}{2}\sigma, \tag{S8}$$

where σ is the Lennard–Jones size parameter of the bead. All SASA calculations were performed using the command:

```
gmx sasa -s mol.pdb -o sasa.xvg -probe <probe> -ndots 4800
```

where `-probe` specifies the probe radius (0.14 nm for AA and 0.191 nm for CG), and `-ndots` was set to 4800 to ensure sufficient numerical accuracy. The same probe size was consistently used within each representation, and comparisons between AA and CG models were performed following these standardized settings.

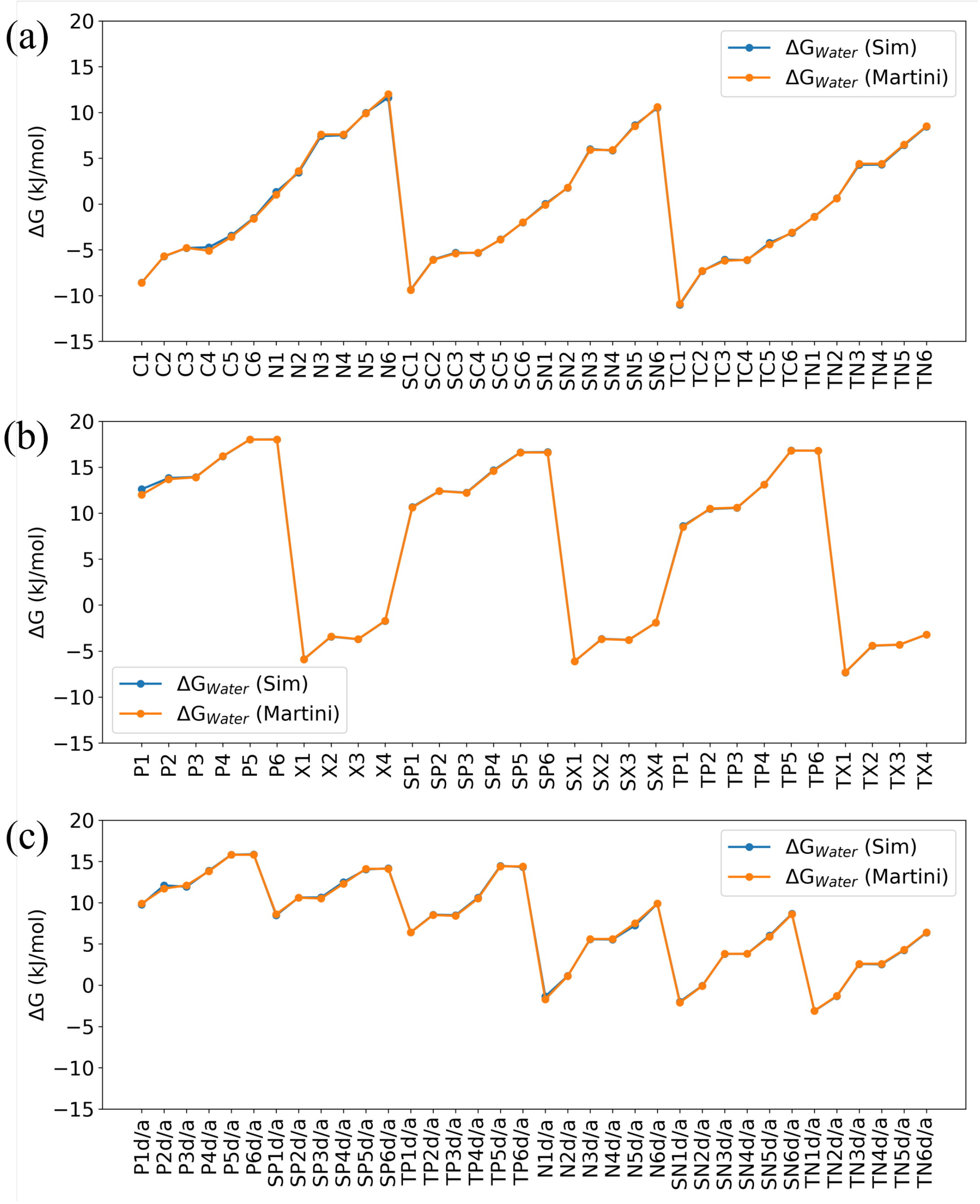

Figure S1: Hydration free energies (ΔG_{water}) of the set of 106 Martini 3 beads in (a)-(c). Results obtained from our TI simulations (blue) are compared against the reference values reported in the Supporting Information of Martini 3 (orange).[2] Each bead represents a distinct chemical motif.

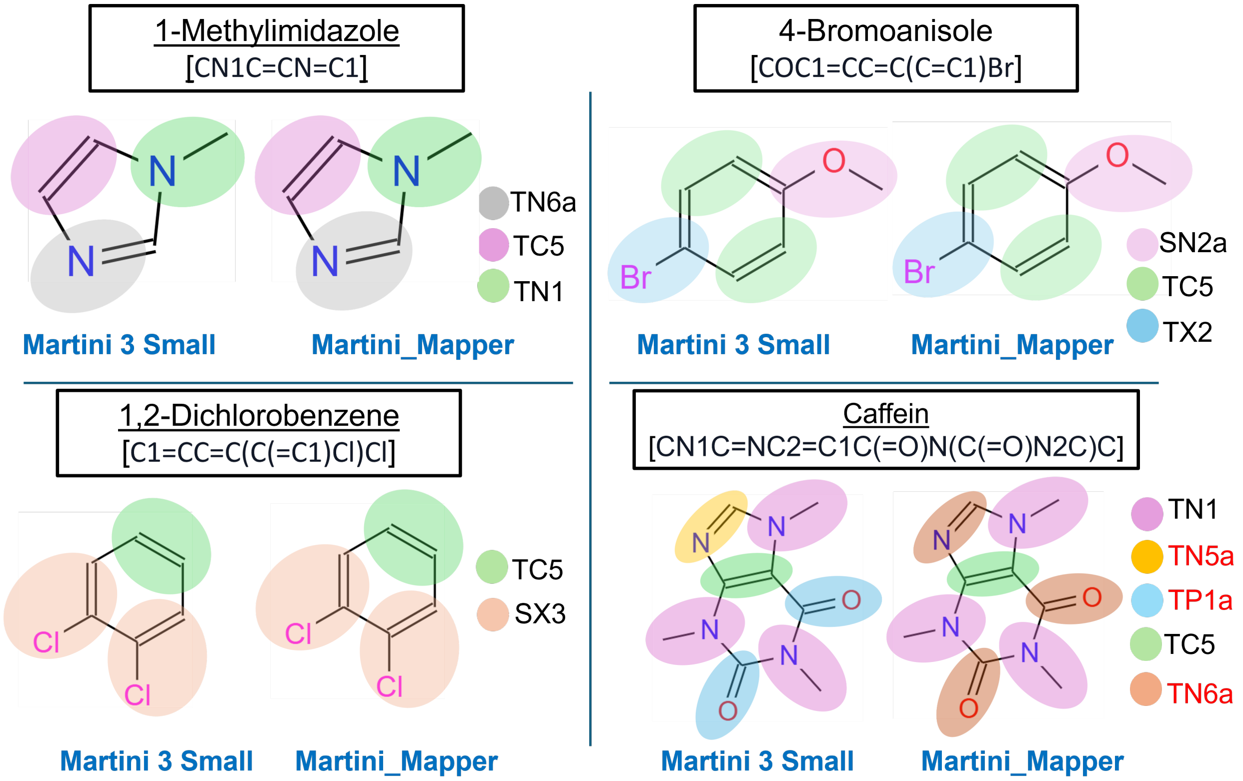

Figure S2: Comparison of the mapped beads of four representative small molecules from the human-made model and generated by the Martini_Mapper. Three molecules, 1-Methylimidazole, 4-Bromoanisole, 1,2-Dichlorobenzene, can be mapped exactly by our Martini_Mapper as the same as the human-made model,[3] while the Caffein model differs by three beads.

References

(1) Pereira, G. P.; Alessandri, R.; Domínguez, M.; Araya-Osorio, R.; Grünewald, L.; Borges-Araújo, L.; Wu, S.; Marrink, S. J.; Souza, P. C. T.; Mera-Adasme, R. Bartender: Martini 3 Bonded Terms via Quantum Mechanics-Based Molecular Dynamics. *Journal of Chemical Theory and Computation* **2024**, *20*, 5763–5773.

(2) Souza, P. C. T. et al. Martini 3: a general purpose force field for coarse-grained molecular dynamics. *Nature Methods* **2021**, *18*, 382–388.

(3) Alessandri, R.; Barnoud, J.; Gertsen, A. S.; Patmanidis, I.; de Vries, A. H.; Souza, P. C. T.; Marrink, S. J. Martini 3 Coarse-Grained Force Field: Small Molecules. *Advanced Theory and Simulations* **2022**, *5*, 2100391.

(4) Durham, E.; Dorr, B.; Woetzel, N.; Staritzbichler, R.; Meiler, J. Solvent accessible surface area approximations for rapid and accurate protein structure prediction. *Journal of Molecular Modeling* **2009**, *15*, 1093–1108.
